# Why might animals remember? A functional framework for episodic memory research in comparative psychology

**DOI:** 10.3758/s13420-024-00645-0

**Published:** 2024-09-17

**Authors:** Alexandria Boyle, Simon A. B. Brown

**Affiliations:** 1 London School of Economics and Political Science, London, UK; 2 CIFAR Azrieli Global Scholars Program, London, UK

**Keywords:** Episodic memory, Hippocampus, Animal cognition, Evolutionary psychology, Comparative cognition, Animal minds, Biological functions, Functions of episodic memory

## Abstract

One of Clayton’s major contributions to our understanding of animal minds has been her work on episodic-like memory. A central reason for the success of this work was its focus on ecological validity: rather than looking for episodic memory for arbitrary stimuli in artificial contexts, focussing on contexts in which episodic memory would serve a biological function such as food caching. This review aims to deepen this insight by surveying the numerous functions that have been proposed for episodic memory, articulating a philosophically grounded framework for understanding what exactly functions are, and drawing on these to make suggestions for future directions in the comparative cognitive psychology of episodic memory. Our review suggests four key insights. First, episodic memory may have more than one function and may have different functions in different species. Second, cross-disciplinary work is key to developing a functional account of episodic memory. Third, there is scope for further theoretical elaboration of proposals relating episodic memory to food caching and, in particular, future-oriented cognition. Finally, learning-related functions suggested by AI (artificial intelligence)-based models are a fruitful avenue for future behavioural research.

## Introduction

The centrality of episodic memory to the human mind invites two questions: What is its distribution, i.e. what forms of episodic memory appear in which species? And what is its function, i.e. why do any of us – human or nonhuman – have episodic memory at all? Clayton and colleagues helped establish some of the most promising approaches to studying the first question through their pioneering work with scrub jays, which inspired work in many different species (for reviews, see Boyle & Brown, In Prep.; Davies & Clayton, 2024; Templer & Hampton, 2013; Zacks et al., 2022). Arguably, they did so by considering a possible answer to the second question: perhaps a function of episodic memory is helping food-caching animals to retrieve the right kinds of food at the right times.

Here, we deepen this insight, by considering possible functions of episodic memory head on, showing both how work on these possible functions has already led to a great deal of insight into the nature and distribution of episodic memory, and, crucially, developing a philosophically grounded framework for thinking about these questions. Our framework suggests four key insights, each with important implications for future research: First, episodic memory may have more than one function and may have different functions in different species. Second, an interdisciplinary approach is key to developing a functional account of episodic memory. Third, there is scope for further theoretical elaboration of proposals relating episodic memory to food caching and, in particular, future-oriented cognition, which have been the subject of significant empirical work. Finally, learning-related functions recently elaborated in philosophy and supported by AI (artificial intelligence)-based modelling are a highly promising avenue for future behavioural research.

### What does ‘function’ mean?

Many biological traits can be understood in terms of their functions – roughly speaking, in terms of what they’re ‘supposed’ to do. The philosophical literature distinguishes two distinct but compatible senses of function, which we can think of as answers to two different explanatory questions (Neander, 2016; Schwartz, 2020).

The first is *causal role function*. Giving an account of the causal role function of a trait involves explaining how it makes a difference to the operations of the systems it is a part of (Craver, 2001; Cummins, 1975). The second is *etiological function.* Etiological functions answer the question: how did this trait *get here*, and why is it *like this*? Evolutionary functions are one sort of etiological function, explaining the existence and nature of traits in terms of natural selection. The most prominent accounts define a trait’s evolutionary function in terms of effects it had in the past which caused it to be selected on (Godfrey-Smith, 1994; Neander, 1991, 2016). Some etiological functions are not evolutionary, however: some traits, including those Heyes (2018) describes as ‘cognitive gadgets’, may have developmental functions, acquired through learning (Garson, 2012; Shea, 2018).

In comparative cognition, ‘function’ is often used in the etiological evolutionary sense. One important goal of comparative cognition is understanding the evolutionary history of cognitive traits. Hypotheses about evolutionary function also play a methodological role in the design of behavioural experiments (see *Why is evolutionary function relevant to investigating the distribution of episodic memory* below). In line with this, we’ll primarily have the evolutionary sense in mind in what follows, unless otherwise specified. Nevertheless, considering hypotheses about the evolutionary origins or phylogenetic distribution of cognitive traits requires attending to causal role functions and developmental functions.

First, it is important to remember that some traits may have developmental rather than evolutionary functions. For instance, humans have brain mechanisms that are specialised for reading, but the written word emerged too recently for this to be due directly to natural selection (Dehaene et al., 2015; Heyes, 2018). Of course, developmental selective processes must have acted on pre-existing neural mechanisms that were themselves most likely the products of natural selection – for instance, mechanisms specialised for pattern recognition. Evolutionary and developmental selective processes interact in complex ways, especially with respect to cognition (Stotz, 2014). The crucial point, however, is that some aspects of episodic memory may only arise – let alone take on certain functions – in specific developmental contexts. Aspects of episodic memory which have developmental rather than evolutionary functions may not be widely shared or have deep phylogenetic histories. As such, developmental and evolutionary functions license different predictions about which species are likely to share traits with us, and about how and when these traits are likely to manifest.

Second, causal role functions are relevant to identifying evolutionary functions, provided we exercise appropriate caution. We cannot read a trait’s evolutionary function directly from its causal role function. Traits may make causal contributions thanks to developmental processes or non-selective evolutionary processes rather than natural selection. Furthermore, traits may have conferred fitness benefits in the past which they no longer produce. Additionally, complex traits are likely to have emerged gradually: at different times, they may have exhibited intermediate forms with different functional profiles, and as a result may have different evolutionary functions in different species (see Box 1). However, understanding a trait’s current role in mechanisms of which it is a part helps us construct hypotheses about selection pressures shaping it in the past: if it played a similar causal role function in the past, as part of a mechanism that responded to an important selective pressure, this could have been amongst its most important evolutionary drivers (see *Future-oriented cognition* and *Learning* sections below).

### Why is evolutionary function relevant to investigating the distribution of episodic memory?

Understanding episodic memory’s evolutionary function(s) is challenging (see Box 2). But it is also crucial to determining its phylogenetic distribution. The core reason why is that it is most productive to search for mental states in contexts where they are most likely to be expressed.

We cannot simply look and see whether a given species has episodic memory in the way that we can discover its anatomy: we must infer episodic memory indirectly from species using it in specific tasks. Even more challengingly, we can only infer its absence from a species’ failure to use it in tasks where we can be confident that they would have used it if they could. Finding tasks where animals are likely to use cognitive capacities can mean combining observation of wild animals with more controlled experiments (Clayton, 1999; Halina, 2023, Sect. 1.4). But it centrally requires looking for cognitive traits in contexts for which they may have been selected. Experiments in artificial settings certainly have a role to play (see below). However, in ‘unnatural’ contexts, animals with a capacity which *could* be used to solve a task may nevertheless not realise that they can do so, may be slow to do so, or may have impediments to doing so which are not immediately obvious. In one classic example, gibbons perform poorly relative to other apes on tasks requiring them to pick up a stick to drag food towards them, which might be thought to reflect fundamental cognitive limitations, for example in causal understanding. Yet it does not. In the wild, gibbons spend much of their time in trees and rarely pick things up off the ground – indeed, their hand shape makes picking things up off the ground difficult. In comparable tasks involving manipulating objects at *shoulder level*, gibbons were on a par with other apes after all (Beck, 1967). Many of the best examples of experimental work informed by such considerations relate to memory and timing capacities as used in foraging (Healy & Hurly, 2013), navigation (Cartwright & Collett, 1987; J. L. Gould, 1986), and food caching (Krebs et al., 1989; Sherry, 1984; Sherry & Vaccarino, 1989; Sherry et al., 1989). Clayton and colleagues’ work on scrub jays sits firmly within this tradition.

While knowing the function(s) of episodic memory would be helpful to searching for its precise phylogenetic distribution, this does not mean that we must answer the function question *first*. Studying the evolution of cognition is notoriously difficult, with a limited fossil record on which to draw and the lure of ‘just so stories’ especially strong. Indeed, one important source of evidence for a trait’s function is typically to be found in its phylogenetic distribution. That is, we typically ascertain why a trait evolved partly by studying which species have it and which species do not, and how their natural histories otherwise compare. Indeed, Clayton and colleagues have powerfully advocated for the role of comparative analysis of which species express episodic memory as a key tool in uncovering its evolutionary history (Clayton & Krebs, 1994; Schnell, et al., 2021a; Van Horik et al., 2012). This seems to imply a puzzling circle: we need to know episodic memory’s function to systematically search for it and establish its distribution, but we need to know its distribution to establish its function.

This issue demonstrates that whilst evolutionary functions are key to investigating episodic memory in animals, they must be treated with care. Proposals about the function of episodic memory are simply hypotheses, often with limited evidence in their support. Even where we are confident that episodic memory has a certain function in one species, we should be cautious in assuming that any state that fulfils that function in any species is episodic memory. It is possible that different traits fulfil similar functions in different species: there may be a variety of strategies underpinning food-caching in diverse species, for instance. Similarly, we should not conclude from finding that an animal is incapable of some function for which episodic memory is sometimes used, that that animal does not have episodic memory: episodic memory could have multiple functions and only perform some of them in some animals (see Box 1).

Yet despite these cautionary notes, pursuing the function and distribution questions in tandem promises progress on both questions. The circularity as formulated above is not cast in iron: we do not need to *know* function to guide our investigation of distribution, or vice versa: we just need reasonable hypotheses. We should work on both questions simultaneously, with tentative answers to one informing and constraining tentative answers to the other, iteratively giving rise to a better overall understanding. The rest of this review emphasises where progress has already been made, and where there are opportunities for further insights.

### Possible functions of episodic memory

Many possible functions have been proposed for episodic memory. In this section we review some possible functions, as well as highlighting the possibility that episodic memory has more than one function, or no function at all.

Some proposed functions are highly specific to particular species’ needs, such as the need for polygynous species like meadow voles to track the reproductive state of mates spread over a large territory, or brood parasitic birds’ need to remember when and where potential hosts have begun building nests (Clayton et al., 2001). Most notably, episodic memory may enable food-caching species to rapidly encode and later retrieve information about the location, status and type of food caches (see below).

Episodic memory may well also have social functions for highly social species. Mahr and collaborators argue that episodic memories enable humans to represent and communicate the reasons for our beliefs, and that this may be among its evolutionary (Mahr, 2022; Mahr & Csibra, 2018) or developmental (Mahr et al., 2023) functions. Davidson et al. (2012) propose that episodic memory may function as ‘social glue’, enabling us to form and maintain important social relationships, and Keven (2024) argues that episodic memory may have played a role in the evolution of human cooperation. Related proposals have been made about episodic memory’s functions in nonhuman social species: alliance formation is important in many non-human primates and dolphins, and episodic memory may provide useful information about potential allies’ past behaviour (Clayton et al., 2001; Davies et al., 2022).

Some proposed functions instead cast episodic memory as a component in more general cognitive processes. One prominent idea highlights episodic memory’s role in imaginatively simulating possible future events (see below). On one version of this view, episodic memory is a *design feature* of future-oriented simulation, providing the raw materials out of which such simulations are constructed. An alternative but related view is that episodic memory is a spandrel, having arisen as a non-adaptive *by-product* of future-oriented simulation (Schulz & Robins, 2022). The simplest version of this account has it that episodic memory still has no function, but episodic memory may instead have first arisen as a by-product but subsequently gained functions of its own (see Box 1).

Others argue that episodic memory is adaptive by virtue of its more ‘backward-looking’ features, highlighting in particular its contributions to various types of learning, including retroactive (Boyle, 2019), unrestricted (Brown, 2024), continual (McClelland et al., 1995) and one-shot (Blundell et al., 2016; Lengyel & Dayan, 2007) learning. Others propose that episodic memory plays an adaptive role in information retrieval (Boyle, 2021). We discuss such learning-related proposals in more detail in *Learning* below.

An important question is how these proposed functions might relate to one another. Episodic memory may have multiple functions, with some being older than others (Box 1). Moreover, context-specific functions and more general functions may be related: for instance, food-caching may be aided by general-purpose learning and future planning. At present, no functions have been conclusively established for episodic memory, meaning that all proposed functions represent live possibilities about its evolution – as does the suggestion that episodic memory is merely a by-product. Exploring these possibilities, along with the relationships between proposed functions, is an important avenue for future work.

Proposals about episodic memory’s function closely connect to a distinct question: what is the function of the *hippocampus* (see Box 2)? The hippocampus is a core part of the neural circuitry underlying episodic memory in humans, and it is known to be involved in tasks suggestive of episodic memory in other mammals and birds (see *Food caching* below and Box 3). However, episodic memory and the hippocampus are distinct: other areas are crucially involved in human episodic memory, including a broad network of neocortical areas (see Eichenbaum, 2017a, and Murray et al., 2017, for overviews). Furthermore, the hippocampus is involved in many capacities which do not obviously involve episodic memory, thanks to its structural features as a key hub network, with inputs from and outputs to different sensory modalities, regions associated with reward/value/valence signals, and frontal regions (Allen & Fortin, 2013; Murray et al., 2017; Zacks & Jablonka, 2023), and dense recurrent connections (Sammons et al., 2024).

Episodic memory may be entirely independent of these other hippocampal functions, or these functions may simply have similar ‘hardware’ needs and hence share neural resources. But two further possibilities are noteworthy. Episodic memory may make use of these other capacities. If so, this could be crucial to understanding the context in which episodic memory emerged: if it mostly used capacities that were already in place, this could mean that it was particularly evolvable, or required relatively little new investment in new machinery. Alternatively, episodic memory may contribute to these other functions in various ways (Cheng & Werning, 2016; Huber, 2024; Schacter et al., 2007). If so, such contributions would be additional functions of episodic memory.

Either way, studying the hippocampus and its functions is crucial to understanding episodic memory and its functions. This is not least because, while brain tissue does not survive in the fossil record, we can have a better idea of the history of the hippocampus than we can of a psychological-level capacity. It is more straightforward to study the morphology of the hippocampus across multiple species, including its embryological development and relevant underlying genes, which facilitates developing detailed accounts of hippocampal evolution. For example, it is likely that all vertebrates have a hippocampus or homologue, whereas other areas, especially granular prefrontal cortex, are much more recent. Relatedly, hippocampal connections to other brain areas may vary significantly across species. For example, the mammalian hippocampus receives significant input from higher-order association areas in the cortex. In the avian brain, higher-order association is primarily carried out in the dorsal ventricular ridge, and this is less densely connected with the hippocampus (Rattenborg & Martinez-Gonzalez, 2011), perhaps indicating that some sensory modalities, abstract representations and higher-order control are less involved in avian memories. If so, a potential further implication is that episodic memory functions differently and/or is experienced differently by birds. Similar points might be made about animals whose brains differ in other ways.

Such physiological facts have been used to support divergent views about how ancient episodic memory itself is, given differing views of exactly which other neural structures are required, from claims that episodic memory is ancient (Allen & Fortin, 2013), to the view that is unique to primates or even humans (Murray et al., 2017), to the view that it has evolved convergently in different lineages (Zacks et al., 2022). Indeed, some behavioural evidence suggests episodic memory may be present in cuttlefish, which lack a hippocampus (Jozet-Alves et al., 2013; Schnell et al., 2021a, 2021b).

### Four questions about three functions

Different proposals about the function of episodic memory have received very different kinds of treatment, from work in a range of disciplines (see Box 3). Some have directly inspired experimental work but leave open important questions that require further theoretical elaboration, while others have received complex elaboration but have not been directly tested. We propose that the function (or potential lack thereof) of episodic memory and its distribution should be investigated in parallel, rather than implicitly treating the question of function as settled or easily resolved. Researchers should therefore elaborate functional hypotheses in appropriate levels of detail and consider how experimental work could test these functional hypotheses as well as hypotheses about episodic memory’s distribution in other species.

To illustrate, we review three of the most prominent proposed functions according to their answers to the same four questions: (1) Why might episodic memory help with securing the proposed adaptive advantage? (2) Which aspects of episodic memory are relevant to this function? (3) What evidence is there, and what evidence might we hope to find in the future, relating to episodic memory’s performing this function in animals? (4) Which aspects of episodic memory might be irrelevant or even counterproductive to this function? By considering proposed functions in this way, we can identify areas where significant work has already been done, and where there are promising avenues for future work. Our aim here is not to endorse any of these prominent accounts of episodic memory’s function, but to highlight that more work is required to fully articulate and evaluate these hypotheses.

#### Food caching

##### Why might episodic memory help with food caching?

If you need to locate your car, you might consult episodic memories of parking it. If you had hidden thousands of seeds, nuts and insects in different locations, and needed to find a particular seed, an episodic memory of caching it might be similarly helpful. Clearly, humans do not use episodic memory *solely* for remembering stored food; but it may be that this is nonetheless amongst the functions of episodic memory. Indeed, one speculative possibility is that this may have been a primary driver of the initial evolution of episodic memory.

##### Which aspects of episodic memory are relevant to food caching?

Several aspects of episodic memory are especially relevant here. One is that episodic memories typically include rich sensory, contextual, and especially spatial information. This includes spatial information about the spatial structure of a recalled scene, tied to many sensory and contextual details that can help locate a cache precisely (e.g., the look of the rock under which you hid that seed, the other side of a gnarled oak from the brook). Episodic memories also typically include information about the location of the event relative to other known locations; indeed, location is so prominent that it is frequently an important way in which episodic memories are accessed. Another reason to think that episodic memory is especially tied to such spatial information is neural (see Box 2).

Episodic memory also frequently includes temporal information. Most relevant to food caching, we often have a sense of how long ago remembered events occurred. This could be especially important when foods decay, ferment or ripen, such that they are particularly valuable at certain durations since caching. This also suggests a use for another feature of episodic memory: keeping track of particular events rather than more general information about, for example, the locations of caches. Remembering caching particular seeds might be a good way of tracking how long ago they were cached.

##### What evidence is there concerning episodic memory and food caching?

Many birds can remember many food caches, and like human episodic memory, this ability depends on the hippocampus (Chettih et al., 2024; Krebs et al., 1989; Sherry, 2011; Sherry & Vaccarino, 1989; Sherry et al., 1989) (see Box 2). Clayton and colleagues’ ground-breaking behavioural work probed this ability, showing that it flexibly represents information about *what* was cached, *where*, and *when*. Clayton and Dickinson (1998) manipulated where jays were able to cache different kinds of food at different times and monitored their retrieval behaviour, revealing that they preferred to uncover recently buried wax worms over peanuts, but peanuts over wax worms buried long enough to decay. This implies that cache recovery is sensitive to how long different kinds of foods have been buried in different locations. This behaviour is flexible: scrub jays update their predictions about whether a food item has decayed when they discover other foods of that kind have decayed at an unexpected rate (Clayton et al., 2003), and can learn to search for wax worms after a long but not short interval (de Kort et al., 2005). Other contextual information can also be represented, such as who was watching at the time of caching (Dally et al., 2009). While the success of these experiments did not require that food caching is a primary evolutionary driver for the development of episodic memory in birds, this is a concrete case in which thinking about the potential adaptive benefits of a cognitive capacity inspired a productive line of behavioural research.

This line of investigation inspired a wide range of studies on many non-avian species, looking for what-where-when memory in food caching and other contexts, including (but not limited to) rodents such as rats, where both positive and negative results were found (Bird et al., 2003; Zhou & Crystal, 2009); Yucatan minipigs (Kouwenberg et al., 2009); dogs (Kaminski et al., 2008); zebrafish (Hamilton et al., 2016); and even cuttlefish (Billard et al., 2020; Jozet-Alves et al., 2013; Schnell et al., 2021a, 2021b) – although it should be noted that details of the experimental paradigm, and hence its interpretability, vary across species. Two recent developments stand out, however. First, Chettih et al. (2024) carried out multi-unit recordings from chickadee hippocampi while they cache and retrieve food. One striking finding is of ‘bar codes’: unlike well-known ensembles of place cells, which have similar patterns of activity coding for nearby places, these patterns function as sharply distinct, unique identifiers for particular caches, and activate only during caching and retrieval. This suggests a unique mechanism is involved above and beyond general spatial cognition. Second, increasingly detailed computational models of scrub jay behaviour have been developed. Brea et al. (2023) recently developed a specialised artificial neural network which can reproduce scrub jay caching and retrieval behaviour across a wide range of tasks, although intriguing questions can be asked about its interpretation (Boyle & Brown, In Prep.).

##### Which aspects of episodic memory might be irrelevant or counterproductive for food caching?

Insofar as episodic memory is thought to involve an elaborate phenomenology known as ‘autonoesis’, such experiences do not seem to have an obvious application to finding buried seeds. Insistence on the importance of autonoesis has led various scholars, most prominently Tulving (2005), to argue that animals only have episodic memory if they have such phenomenology. This in turn has led researchers who have found evidence of rich forms of memory for food caches, but without direct evidence of the associated phenomenology, to avoid attributing ‘episodic memory’, instead restricting themselves to terms like ‘episodic-like memory’ (Clayton & Dickinson, 1998; Feeney et al., 2009; Zinkivskay et al., 2009). This terminological shift is commonly justified on the grounds that phenomenological features of episodic memory cannot be detected behaviourally. However, there are objections both to treating human-like phenomenology as necessary for episodic memory, and to the idea that animals’ phenomenological experiences cannot be behaviourally detected (see Boyle, 2020; Boyle & Brown, In Prep.; Eichenbaum et al., 2005).

Irrespective of issues concerning consciousness, other features of episodic memory seem unhelpful for food caching: while no doubt many details about a food cache are or could be useful to recall, many are not. And while details about landmarks, such as the shape of a rock, might aid in locating caches precisely, it may be just as helpful to recognise landmarks when confronted with them, without any associated ability to ‘replay’ them offline when far away.

A related issue is that episodic memory manifests in a wide range of contexts beside food caching, which a food caching function would not predict. In addition, there is evidence for episodic memory in many non-caching species, such as dolphins (Davies et al., 2022). Again, this is not predicted by a food-caching function. The force of this observation turns on how we spell out the food-caching function: is episodic memory envisaged as a domain-specific adaptation that only supports food caching, or is food caching simply an application of a domain-general mechanism which is highly adaptive in certain species? Different answers to this question would support different predictions about when episodic memory should manifest. For example, a more domain-general capacity might be more likely to perform other evolutionary or developmental functions in addition to food caching, and to manifest in ‘unnatural’ experimental settings.

A further issue is whether the kinds of temporal representation associated with human episodic memory would help with recovering fresh caches. Hoerl and McCormack (2017, 2019) suggest allegedly simpler mechanisms would suffice. However, it is not clear that their proposed mechanism explains the kinds of flexibility with respect to time found in cache memories (Clayton et al., 2003; de Kort et al., 2005), and the philosophical interpretation of their models, including the idea that they are simpler in a relevant sense, is more complex than first appears (Brown, 2023).

Finally, it is worth noting that not all food caching and recovery strategies require anything like episodic memory: for example, if an animal is simply more likely to visit certain locations for independent reasons, they will tend to both cache and search for caches in those locations irrespective of whether they remember the caches at all (Applegate & Aronov, 2022). Episodic memory need only be useful in situations like those explored by Clayton, where features of caches like type of food are worth remembering.

#### Future-oriented cognition

##### Why might episodic memory help with future-oriented cognition?

Humans can take the ‘long view’ when making decisions, taking into account predicted future consequences. We delay gratification, engage in complex, temporally extended projects with minimally immediately rewarded steps on the promise of an eventual reward, and anticipate future preferences which will diverge from current ones. It has been proposed that episodic memory plays an important role in this kind of future-oriented cognition.

One way episodic memory might support this is by our learning from episodic memories of relevantly similar events to inform predictions and act accordingly. We return to these ideas in the next section, *Learning*. More commonly, future-oriented accounts of episodic memory’s function suggest that episodic memory plays a role in our ability to engage in *future-oriented simulation*: imaginative projection into the future.

One oft-repeated motivation for this view is that ‘the adaptive advantage of any memory system can only lie in what it contributes for future survival’ (Suddendorf & Corballis, 2007). It should be stressed, though, that this observation falls short of motivating the view that episodic memory’s function involves future-oriented cognition. All adaptive cognitive traits must drive behaviour that pays off over time, but they need not do this by being ‘about’ the future in any literal sense. A further motivation is the apparent difficulty of articulating how episodic memory’s ‘backward-looking’ operations might generate adaptive behaviour (Hoerl & McCormack, 2016; Suddendorf & Corballis, 2007). Again, more on this in the next section (*Learning*).

As noted above, episodic memory is construed variously as a design feature or functionless by-product of future-oriented simulation.

On the ‘by-product’ view, episodic memory arises as a natural by-product of a system producing future-oriented simulations; these are different manifestations of the same cognitive process. This view fits with the idea that episodic memory involves ‘simulating past events’. Against this, Robins (2022) argues that episodic memory and future simulation are distinct cognitive processes with different principles of operation and distinct causal role functions.

On the ‘design feature’ view, episodic memories provide the ‘raw materials’ out of which future-oriented simulations are constructed: we simulate future events by recombining elements of our episodic memories in novel ways (Schacter & Addis, 2007; Suddendorf & Corballis, 2007). An important question for the ‘raw materials’ idea is why such raw materials would be needed. The idea that imaginative experiences must be constructed out of previous experience is old but controversial, going back at least to Hume (1748) (see Van Leeuwen, 2013, for discussion). There is some evidence that this is indeed an aspect of episodic memory’s causal role function: for instance, future-oriented simulations are experienced as more vivid and detailed when they include contexts that have recently been experienced (Schacter et al., 2007). But it is a further question why a future-oriented simulation system should rely on *episodic* memory in this way, rather than being trained on experiences as they occur, with components of experienced events being stored separately, ready for recombination, rather than in full episodes. Furthermore, the computational mechanisms of extraction of features from episodic memories and their recombination are rarely articulated. As a result, the view that episodic memory supports simulation by providing its raw materials is underspecified in a way that makes it impossible to robustly evaluate. Spelling out this hypothesis in more detail is an important avenue for future work.

Future-oriented accounts also owe an account of why we simulate the future, rather than considering it abstractly. Future-oriented simulation is generally thought to be adaptive because it enables organisms to plan for the future, engage in goal-oriented behaviour requiring several steps, anticipate future rewards and harms, and anticipate how future preferences might diverge from those in the present (Suddendorf & Corballis, 2007). But just as accounts of episodic memory must address why it stores integrated memories for particular events in context rather than decontextualised information, we might ask why organisms plan for the future and so on through simulating particular possible future scenarios rather than through using more general models and rule-based approaches to prediction. One proposal is that simulation provides a direct emotional engagement with future events, increasing their motivational force and counteracting a tendency to disregard temporally distant future events (Boyer, 2008). However, it is currently uncertain exactly how episodic future thinking relates to delay discounting, with some results supporting this possibility and others suggesting otherwise (Ye et al., 2022); for example, episodic amnesics perform similarly to healthy controls in temporal discounting despite being unable to engage in future-oriented simulation (Kwan et al., 2013).

Whilst there is some evidence for a connection between episodic memory and future-oriented cognition (see *What evidence is there concerning episodic memory and future-oriented cognition*), the puzzles discussed in this section highlight areas where further theoretical work would be useful. In particular, an account spelling out more precisely how future-oriented cognition relies on episodic memory and why it takes the form it does is needed. A potential solution to both problems is to posit that episodic memory emerged independently for a different function and was later recruited to support future-oriented simulation. This might explain why it was available to be recruited for imagination in the first place, and why we use future-oriented simulation: it might be that the latter took on the rich, contextual character of episodic memory *because it was co-opted from episodic memory*, not because of intrinsic advantages over other potential forms of future-oriented cognition. This possibility should motivate giving greater consideration to other, perhaps older, functions of episodic memory.

##### Which aspects of episodic memory are relevant to future-oriented cognition?

A precise account of exactly which features of episodic memory are relevant to future-oriented cognition can only be given in the context of a theory of precisely how and why future simulation draws on episodic memory. However, we can point to various features of episodic memory that seem promising.

First, episodic memory’s rapid storage of large amounts of detail may support future-oriented simulation by providing a rich, varied bank of raw materials for future-oriented simulations, including structural information about the ways in which various components have been combined in the past. Second, episodic recollection and future-oriented simulation share neural underpinnings (Box 2), are phenomenologically similar, and may rely on similar computations (Addis, 2018; but see Robins, 2022). As such, episodic memory’s format may suit it particularly well to being re-used for simulations. Finally, both episodic memory and simulation seem to rely on constructive mechanisms and may both be optimized to allow flexible recombination of different elements from stored information. It has been proposed that this could explain various memory errors: because retrieval involves reconstruction drawing on a range of processes and background knowledge, it does not result in perfectly faithful representations of events (De Brigard, 2014; Michaelian, 2016; Schacter, 1999; Schacter et al., 2011, 2018). For the by-product view, episodic memory is reconstructive simply because it uses simulation machinery selected to produce plausible scenarios rather than to accurately represent the past; for the design feature view, episodic memory does need to provide raw materials for simulations, but again need not do so through accurate representations of the past.

##### What evidence is there concerning episodic memory and future-oriented cognition?

There are several lines of research investigating animals’ capacities for future-oriented cognition.

One turns on intertemporal choice tasks involving delayed gratification, where animals forego immediate reward in favour of larger delayed rewards. However, it has been pointed out that for many species and many environments, foregoing immediate reward is unlikely to be adaptive, insofar as greater rewards in the future are too uneven and stochastic in those ecological niches to be relied upon, so this may not be an ecologically valid method for investigating future-oriented cognition (for review, see Raby & Clayton, 2009; Redshaw & Bulley, 2018).

Sequential tool-use tasks can also probe capacities for complex, temporally extended action. Here, tools must be used to obtain non-food objects, which can themselves be used as tools to obtain food. New Caledonian crows have completed three-step sequential tool tasks (Wimpenny et al., 2009), whilst chimpanzees, orangutans, and bonobos complete tasks with up to five steps (Martin-Ordas et al., 2012). Studies have also investigated the ability to acquire tools to solve anticipated future problems. In one relevant study, chimpanzees and orangutans were found to select tools which could be used to obtain a reward in another room at a future time, in some cases preferring the tool to an immediate reward and selecting the correct tool from a range of options even where it had not been encountered before (Osvath & Osvath, 2008). A pre-registered study using a similar design found that New Caledonian crows could also select appropriate tools for future tasks, preferring these to immediate food rewards and to tools that had been more strongly associated with reward in the past (Boeckle et al., 2020). Observations of wild chimpanzees also suggest they plan tool use for foraging at termite nests, arriving at the nest with tools appropriate to the situation, including sets of different tools to be used in sequence (Musgrave et al., 2023). Captive chimpanzees have been observed storing piles of rocks in the morning before throwing them at zoo visitors later in the day (Osvath, 2009; Osvath & Karvonen, 2012). Chimpanzees will also construct tools which they will later have the chance to use to obtain rewards, though have difficulty preparing the number of tools that will be required (Bräuer & Call, 2015).

Another prominent line of evidence relates to whether other animals can plan for their future preferences diverging from their current ones. The Bischof-Köhler hypothesis postulates only humans can do this (Suddendorf & Corballis, 1997). However, Raby et al. (2007) found that when not hungry, scrub-jays preferentially cache foods in locations where they expect to be hungry in the future, preferentially storing seeds in locations they had previously only been fed dog kibble, and vice versa. By contrast, similar studies with Canada jays and Eurasian jays did not find this pattern of behaviour (Amodio et al., 2021; Martin et al., 2021). Whilst this may raise questions about the interpretation of the scrub-jay results, it may be explained by differences between the species and methods involved. In another study, Correia et al. (2007) used specific satiety to dissociate scrub-jays’ current and future preferences. When fed on one type of food, they become sated on that food and prefer to eat something else. When scrub-jays were given the opportunity to cache two types of food, one of which was provided prior to caching and the other prior to recovery, they preferentially cached the food they would not be sated on at the time of recovery, despite being sated on that food at the time of caching.

The interpretation of this body of evidence is the subject of considerable debate, including about whether such behaviours might be explained by associative processes, whether they require thinking about the future, and what the answers to these questions imply (Brown, 2023; Hoerl & McCormack, 2019; Redshaw & Bulley, 2018). Perhaps a more important question for our purposes is whether these behaviours provide evidence for episodic memory playing a causal role in future-oriented cognition in animals. The envisaged role for episodic memory is providing the raw materials for future-oriented simulation. However, just as it is difficult to establish that information an animal retains about the past takes the form of *episodic* memory, it would be difficult to show that the behaviours described here involve episodic future simulation rather than, say, semantic thought about the future (Raby & Clayton, 2009). Whereas in the case of episodic memory there may be behavioural signatures that could aid in making that discrimination (Boyle, 2020), it is less clear what the behavioural markers of episodic future simulation might be (against Schulz & Robins, 2022). Establishing behavioural markers of episodic versus semantic future thinking could be an important direction for future work. Moreover, even if it is granted that animals engage in future-oriented simulation, it may be a further question what role, if any, episodic memory plays in this process. Investigating this would require developing a more detailed account of episodic memory’s causal role in future-oriented simulation which makes testable hypotheses, perhaps with the aid of computational modelling.

##### Which aspects of episodic memory might be irrelevant or counterproductive for future-oriented cognition?

In addition to the open questions above about precisely how and why episodic memories are involved in future-oriented simulation, some features of episodic memory seem especially hard to explain if its evolutionary explanation is exhausted by its relationship to future-oriented cognition. For one thing, although much has been made of the systematic errors to which episodic memory is subject, episodic memories are frequently accurate in many respects. If episodic memory is primarily supposed to furnish raw materials for future-oriented simulation, we might expect such accuracy to be rare (Mahr & Csibra, 2018; but see Schacter et al., 2018, for an opposing view). By the same token, episodic memory is ubiquitous (Brown, 2024): we frequently access our episodic memories in a wide range of circumstances besides future simulation. Again, if episodic memory primarily serves to provide raw materials for future-oriented simulation, we might not expect these memories to be so prominent in our mental lives. Finally, episodic memories often carry a temporal component, indicating *when* the remembered event happened. Episodic memory’s role in future-oriented cognition might make it useful to mark simulations according to whether they concern ‘past’ or ‘future’ to avoid confusing them (Michaelian, 2016). However, more granular information about *when* in the past an event occurred would not be required for this purpose. Note that none of these points raises a problem for the idea that future-oriented cognition is *one of* episodic memory’s functions; they do, however, undermine the commonly espoused idea that it is its *only* or *primary* function.

#### Learning

##### Why might episodic memory help with learning?

Intuitively, episodic memory is closely entwined with learning. We often remember the exact circumstances in which we learned some piece of information. Memory of the circumstances of learning can be so bound up with the information learned that we can mentally retrace our steps and use the experience to cue retrieval of the information. We also often learn by reflecting on our past experiences, thinking about what we might have done differently, what lessons can be learned, and so on, as well as gleaning insights from recalling, comparing, contrasting and categorising experiences.

Learning theory, however, especially as applied to animals, rarely gives a central role to episodic memory. Deep learning models and traditional reinforcement learning approaches often imply that subjects learn by incrementally updating their patterns of behaviour in response to new experiences, rather than reflecting on any one event after the fact. This means it is not obvious what exactly the function(s) of episodic memory might be for learning, or how its role in learning might be adaptive. Episodic memory is clearly relevant to learning *about the details of particular past events*, but the evolutionary benefits of this are unclear.

Several possibilities have been developed, however. All give episodic memory an important causal role function (see above) within a broader learning mechanism which may have been selected for. Many revolve around the idea that episodic memory allows for extremely rapid acquisition of information about a particular event, and therefore can supplement incremental (hence slow) kinds of learning.

In ‘episodic control’ algorithms (Lengyel & Dayan, 2007), when faced with a decision, agents remember events involving similar decisions and take whatever action was most successful in those scenarios – although more complex versions allow for different past episodes to be combined (Gershman & Daw, 2017). By contrast, in standard reinforcement learning algorithms, those past successes will have had only a tiny effect on the agent’s value function, and would need to be repeated many times to reliably bring about similar behaviour.

More general kinds of learning from episodic memory are possible, which can be useful for a few reasons. Firstly, the memories can effectively provide extra training data, with more insights being mined from them each time they are replayed. One trick this allows is reassessing the source and significance of old information upon the receipt of new information (Cosmides & Tooby, 2000). In turn, this can allow for the learning of complex patterns which cannot be learned from a single look at an experience, especially in environments with kinds of complexity and instability that traditional machine-learning approaches will struggle to capture (Boyle, 2019; Brown, 2024; Gershman & Daw, 2017). A special form of learning from past experience could involve coming to regret that experience upon reassessing it, and using this reassessment to guide future decision-making that anticipates regret (Hoerl & McCormack, 2016). Secondly, replay of past experiences can allow for interleaved training, in which the learning system is exposed to a variety of different learning events. This facilitates ‘continual learning’, ensuring that new information is gradually incorporated into the system’s broader knowledge structure without overriding existing information (so-called ‘catastrophic forgetting’) (McClelland et al., 1995). Related ideas are developed in Boyle (2021) and Nadel and Moscovitch (1998).

Episodic memory may also have an important function in usefully organising other information, so that the right pieces of information are accessed at the right time. Its specialisation for rich associations and spatiotemporal information may make it well suited for this kind of role, as suggested by the use of ‘memory palace’ techniques for advanced memorisation (Boyle, 2021).

##### Which aspects of episodic memory are relevant to learning?

Which aspects of episodic memory are most relevant depends on which of these proposed learning-related functions we consider. Primarily, these functions draw on episodic memory’s rapidly storing rich structures of information. Several also draw on the fact that episodic memory provides access to particular past events rather than general patterns (even very detailed, specific, but nonetheless repeatable patterns).

For example, episodic control requires recalling sequences of events in enough detail to determine which actions were previously successful in highly specific contexts. Learning from replaying episodic memory requires that each memory includes enough information to be informative, and keeps the specific circumstances of each event distinct. Specific forms of replay-learning impose further requirements: for example, regret might turn on episodic memory having special ties to emotion, both in recalling emotions one was feeling at the time and being able to form new emotions about past events now. Incorporating new information into existing knowledge structures and consolidating it more firmly requires only that episodic memory be as rich as the information that is stored. But gaining new insights from old data or reassessing the circumstances in which information was learned requires making extremely rich information available, especially for discovering complex or subtle patterns that were not noticed at the time of the relevant experiences. Meanwhile, for a role in organisation of stored non-episodic information, episodic memory needs to be not just richly associative but highly structured, with spatiotemporal and other frameworks allowing for reliably stored, complex structures that can be ‘navigated’ to find relevant information later.

##### What evidence is there concerning episodic memory and learning?

Evidence for the use of episodic memory for specific learning algorithms in humans and other animals is harder to come by than for more directly observable behaviours like food caching. Ultimately, it requires developing computational models of learning and fitting them to detailed neural and behavioural data.

Algorithms using states that are in some ways akin to episodic memory are beginning to be developed and tested in detail (see Boyle & Blomkvist, 2024, for review). For example, Mnih et al. (2015) develop a reinforcement learning algorithm with a kind of replay which excels in video game performance relative to more standard RL algorithms, and Zeng et al. (2023) compare three RL algorithms with different memory structures: a standard RL algorithm without anything like episodic memory; an algorithm using offline replay to drive learning; and an algorithm inspired by episodic control. Zeng et al. find, in line with theoretical predictions, that the ‘episodic memory’-based algorithms learn faster (i.e., with fewer new experiences) than algorithms without it, with episodic control algorithms learning fastest but tending to explore less and get stuck at local optima, such that replay-based learning algorithms display better performance in the long run.

If different episodic memory-based learning systems have distinctive costs and benefits, then evolution may favour minds using multiple learning systems, for example relying on fast-learning episodic control style systems early in the learning process and more thorough kinds of learning later on. Different environments may also affect which learning processes are best, including how changeable the environment is and what the costs of failure in an environment may be (Gershman & Daw, 2017).

We expect further work in this vein, incorporating more detailed models of episodic memory and other ideas about memory and learning surveyed above, to provide further insights. However, computational modelling on its own does not tell us about which algorithms are in fact used in humans and other animals, and we also expect that as such algorithms are better understood, they will be tested more directly.

There is some evidence that is germane to some of these ideas, if not a direct test of different learning algorithms. One line of evidence relates to hippocampal lesions, which tend to both impair episodic memory and slow learning, whether in humans (O’Kane et al., 2004; Rosenbaum et al., 2005) or rodents (Kosaki et al., 2014; Wiltgen et al., 2006). Another testable prediction of several of the functions of episodic memory for learning canvassed above is that episodic memory should make available information which is not immediately incorporated into broader knowledge structures, including information whose relevance to tasks is not obvious at the time of storage. ‘Unexpected question’ tasks, also known as ‘incidental encoding’ tasks, can be interpreted as tests of this prediction. For example, dolphins can follow unexpected commands to retrieve hidden objects whose locations would not have been salient to them before receiving the command, suggesting incidental encoding of the object’s location (Davies et al., 2022). Such tasks have been administered to a wide range of animals, including pigeons (Zentall et al., 2001, 2008), dogs (Fugazza et al., 2016), rats (Sato, 2021; Zhou et al., 2012), dolphins (Davies et al., 2022) and jays (Davies et al., 2024a, 2024b). Memories of the source of information (e.g., which sensory modality was used) can also be especially useful for various kinds of retrospective learning, and have been found in cuttlefish (Billard et al., 2020).^1^

While learning-based proposals have been articulated in deeper, more fine-grained detail than those relating to future-oriented cognition (see above), in some cases specifying algorithmically how and why episodic memory may contribute to learning, they largely await more detailed and direct tests. Many promising ideas about the uses of episodic memory for learning have not been directly implemented in concrete working algorithms, let alone tested in animals. However, we see the work of developing such algorithms and testing their promise as models of animal cognition, with both neural and behavioural data, as a major promising route for future development in the field.

##### Which aspects of episodic memory might be irrelevant or counterproductive for learning?

While learning-related functions seem to require episodic memory to be rich, rapidly formed, and highly structured, it seems less important that it always involves a distinctive kind of phenomenology. Indeed, those uses that refer to repeated replay for the purposes of consolidating information or reappraising and learning from past experiences seem to require a great deal of episodic remembering, such that much of it would need to be unconscious – including during sleep.

Episodic memory’s reconstructive properties are also not straightforwardly predicted by these accounts. As noted above, these reconstructive properties are thought to be mechanistically connected to episodic memory’s role in simulation and to give rise to memory errors. If episodic memory is unreliable as a guide to details of past events, then it may be a poor basis for learning – indeed, some have argued on this basis that accurately conveying information about the past is not among episodic memory’s functions (see especially De Brigard, 2014). On the other hand, whilst episodic memories are undeniably reconstructive, the prevalence of episodic memory error may be overstated: reconstruction and unreliability are not the same thing. The fact that misremembering can be reliably induced by certain procedures under lab conditions does not show that episodic memory is unhelpful for learning in normal circumstances – just as we use vision for learning in normal circumstances, even though visual illusions can be reliably induced. Indeed, it has been argued that these errors are evidence of mechanisms that, on the whole, result in accurate memories (Michaelian, 2016; Puddifoot & Bortolotti, 2019; Roediger III, 1996). For example, one prominent case of memory reconstruction is the DRM (Deese-Roediger-McDermott) effect, in which subjects attempting to recall a list of words are likely to falsely report having seen words semantically related to those in the list (Deese, 1959; Roediger & McDermott, 1995). Whilst this does imply some memory errors, it is probable that the mechanism responsible also makes it more likely that subjects correctly recall the words they did see. On the whole, in natural settings, this reconstructive mechanism may make memory more helpful for learning and decision making, by increasing the availability of memory content of a tolerable level of accuracy.

## Conclusion

As Clayton’s work shows, it is crucial to consider the function of episodic memory when investigating which animals have it. Like investigating episodic memory in animals, though, developing a functional account of episodic memory is extremely challenging. As a complex capacity, episodic memory is likely to have multiple functions, and may have different functions in different animals. A functional account should consider how these functions relate, both to one another and to functions of the hippocampus, and how they might be distributed across species. Developing such a detailed picture of episodic memory’s emergence over evolutionary time is a demanding task, given the available evidence. As this review illustrates, a cross-disciplinary approach can help meet this challenge: traditional approaches from comparative cognition and neuroscience can be supplemented with both computational and philosophical work.

By surveying three of the most prominent proposed functions for episodic memory in detail, we have highlighted areas where future work of different kinds may prove particularly fruitful. There is an important body of evidence relating to episodic memory and food caching in a number of species. Here, further theoretical elaboration would be useful in articulating the generality of this function, and computational modelling could further specify the underlying mechanisms. Similarly, future-oriented cognition has been extensively investigated in a range of animals, but there are outstanding questions about precisely why and how episodic memory might be involved in future-oriented cognition which raise questions about the interpretation of this evidence. Further theoretical and computational work would be useful here, too. By contrast, learning-related functions for episodic memory have recently been articulated in some detail in philosophy and investigated using computational methods, but are yet to be fully explored in comparative cognition. Further work to empirically investigating these learning-related functions in animals is a significant, highly promising avenue for future behavioural research.

**Table:** 

**Box 1: How many functions?**	
It is common to speak of ‘the function of episodic memory’. However, researchers should be live to the possibilities that episodic memory may have more than one – or, indeed, zero – functions, to avoid unhelpful debates about *the* function.	
Traits commonly evolve in response to multiple selective pressures. This can occur when one innovation has multiple selective advantages from its inception, or when traits initially conferring one advantage later come to be used in other advantageous ways. For example, feathers are useful both for insulation and for flight; both of these have likely shaped the form feathers take today. Similarly, it is likely that episodic memory has survived due to conferring several different advantages: for example, it could have initially evolved for food caching and subsequently taken on broader functions related to learning. Each function may have shaped contemporary manifestations of episodic memory in different ways, including via trade-offs between different functions. Furthermore, different functions may have emerged or been retained in different lineages, leaving episodic memory with different functions in different animals. Finally, episodic memory may have many functions, each conferring only a small selective advantage, but collectively justifying its expense. So it does not follow, for example, from episodic memory’s having a large metabolic cost, that it must have a correspondingly large *single* benefit.	
Some traits have no evolutionary function, arising through developmental or non-selective processes. It is also common for traits to emerge as ‘spandrels’, non-selected by-products of other adaptive traits (S. J. Gould & Lewontin, 1979). As with the architectural feature after which ‘spandrels’ are named, which initially arose as a by-product of building arches but came to be used for elaborate decoration, such non-functional traits may take on functions over time (Godfrey-Smith, 1994). This is also a possible history for episodic memory.	

**Table:** 

**Box 2: Possible functions of the** ***hippocampus***	
The hippocampus is involved in numerous processes, and likely has many functions, several of which overlap. Some of the most widely discussed include the following:	
The hippocampus is involved in forming rich and complex associations, including cross-modal associations combining different sensory modalities and streams of information, and associations involving complex structures (Allen & Fortin, 2013; Henke, 2010; Zacks et al., 2022), although see Rattenborg and Martinez-Gonzalez (2011). It is also crucial to rapid, flexible learning, including trace conditioning (associations between stimuli separated in time) (Bangasser et al., 2006; Henke, 2010). In some taxa it may play additional roles in integrating and manipulating information which in mammals are performed neocortically – potentially even including orchestrating a ‘global workspace’ (Zacks & Jablonka, 2023).	
Famously, the hippocampus is involved in representing spatial structure, thanks to place cells, which fire when animals are in particular locations. Their overall ensemble seems to support a ‘cognitive map’ in many animals (Bingman & Sharp, 2006; Moser et al., 2008; O’Keefe et al., 1978). However, some have suggested that the hippocampus is specialised for coding abstract structure in general, with space as a special case (Eichenbaum, 2017b).	
The role of the hippocampus in unifying different kinds of information into a representation of the overall current context closely relates to the its role in *event segmentation*, marking boundaries of events (Clewett et al., 2019). This is crucial to episodic memory, but is also important for shorter term memory and learning processes (Kurby & Zacks, 2008). The hippocampus shows a distinctive signal at event boundaries, which seem to reset its representation of context, and is also involved in coding hierarchical structure, with sub-events composed into longer events.	
Another closely related causal role function is in supporting scenario construction or event simulation. Imaging and lesions show that the human hippocampus is involved in simulating possible future scenarios and counterfactual scenarios (*what would have happened if*…), as well as in dreaming (De Brigard & Parikh, 2019; Hassabis et al., 2007; Maguire & Mullally, 2013; Schacter et al., 2015; Spanò et al., 2020). A potentially related phenomenon found in numerous species is ‘replay’, where neural representations rapidly fire in sequences corresponding to past or potential future courses of action: the best-studied instance of replay is in hippocampal place cells (Liu et al., 2022; Ólafsdóttir et al., 2018).	

**Table:** 

Box 3: How are functions investigated?	
Several disciplines are relevant to investigating the functions of episodic memory.	
The emphasis on food caching comes from the tradition of ethology: careful observation of animals’ behaviour in their natural habitat. Important work leading up to Clayton and Dickinson (1998) – reviewed in (Sherry, 2011) – combined such observation with investigation of factors like the size of different birds’ hippocampi. More recent work has also investigated related phenomena in the wild (Davies et al., 2024a, 2024b; Harten et al., 2024; Janmaat et al., 2006a, 2006b). Ethological observation can be supplemented by experimental work, both purely behavioural work like many of Clayton’s now-classic papers on scrub jays, and in combination with neural methods such as optogenetics and electrophysiology (e.g., Chettih et al., 2024; Crystal, 2021; Panoz-Brown et al., 2018; Sato, 2021). In addition to shedding light on the mechanisms underpinning episodic memory, these methods can be especially helpful for discerning the causal role functions of episodic memory, which in turn can support investigations into which causal role functions were selected for.	
Ideally, such observation would be supplemented with direct evidence concerning the ecological demands and opportunities faced by the relevant ancestors of today’s species, and evidence for natural selection (including evidence that variations in the trait have a genetic basis; and that the trait improves reproductive success), or a well-specified and -supported developmental form of selection. In the absence of such evidence, we can make more moderately supported inferences about when certain traits likely arose on the basis of the distribution of the trait of interest today, for example using considerations of cladistic parsimony (Currie, 2021; Halina, 2023; Schnell, Amodio, et al., 2021; Sober, 2005, 2009, 2015; Van Horik et al., 2012).	
Computer science is also useful here, particularly in articulating proposals about episodic memory and related cognitive traits in detail and testing them, as exemplified by Zeng et al. (2023) (see *Learning* section). Computational work can also aid in developing detailed proposals about the mechanisms underlying performance in episodic memory tasks studied in comparative cognition, as exemplified by Brea et al. (2023).	
Philosophy, meanwhile, has multiple roles to play. Work like that of Boyle (2019, 2021), Brown (2024), De Brigard (2014), Mahr and Csibra (2018), Schulz and Robins (2022) and Schwartz (2020) has articulated different possibilities for the evolution of episodic memory with greater precision. Philosophical work has also carefully articulated different understandings of what episodic memory is and what forms it could take in different animals, synthesizing insights from quite different literatures, and worked through thorny methodological issues which are hotly disputed amongst scientists (see especially a review in Boyle and Brown (In Prep.)).	

## Data Availability

Not applicable.

## Footnotes

1 The term ‘source memory’ is also used by Crystal et al. (2013), but arguably with a different meaning. This study shows that rats can remember contextual information about whether they found a location themselves or were placed there by an experimenter.

## References

[CR1] Addis, D. R. (2018). Are episodic memories special? On the sameness of remembered and imagined event simulation. *Journal of the Royal Society of New Zealand,**48*(2–3), 64–88. 10.1080/03036758.2018.1439071

[CR2] Allen, T. A., & Fortin, N. J. (2013). The evolution of episodic memory. *Proceedings of the National Academy of Sciences*, *110*(supplement_2), 10379–10386. 10.1073/pnas.1301199110 10.1073/pnas.1301199110 PMC3690604 23754432

[CR3] Amodio, P., Brea, J., Farrar, B. G., Ostojić, L., & Clayton, N. S. (2021). Testing two competing hypotheses for Eurasian jays’ caching for the future. *Scientific Reports,**11*(1), 835. 10.1038/s41598-020-80515-7 33436969 10.1038/s41598-020-80515-7 PMC7804264

[CR4] Applegate, M. C., & Aronov, D. (2022). Flexible use of memory by food-caching birds. *eLife*, *11*, e70600. 10.7554/eLife.70600 10.7554/eLife.70600 PMC9038193 35467526

[CR5] Bangasser, D. A., Waxler, D. E., Santollo, J., & Shors, T. J. (2006). Trace Conditioning and the Hippocampus: The Importance of Contiguity. *The Journal of Neuroscience,**26*(34), 8702–8706. 10.1523/JNEUROSCI.1742-06.2006 16928858 10.1523/JNEUROSCI.1742-06.2006 PMC3289537

[CR6] Beck, B. B. (1967). A Study of Problem Solving by Gibbons. *Behaviour,**28*(1/2), 95–109. 6016362 10.1163/156853967x00190

[CR7] Billard, P., Clayton, N. S., & Jozet-Alves, C. (2020). Cuttlefish retrieve whether they smelt or saw a previously encountered item. *Scientific Reports,**10*(1), 5413. 10.1038/s41598-020-62335-x 32214190 10.1038/s41598-020-62335-x PMC7096502

[CR8] Bingman, V. P., & Sharp, P. E. (2006). Neuronal implementation of hippocampal-mediated spatial behavior: A comparative evolutionary perspective. *Behavioral and Cognitive Neuroscience Reviews,**5*(2), 80–91. 10.1177/1534582306289578 16801684 10.1177/1534582306289578

[CR9] Bird, L. R., Roberts, W. A., Abroms, B., Kit, K. A., & Crupi, C. (2003). Spatial memory for food hidden by rats (Rattus norvegicus) on the radial maze: Studies of memory for where, what, and when. *Journal of Comparative Psychology (Washington, D.C.: 1983)*, *117*(2), 176–187. 10.1037/0735-7036.117.2.176 10.1037/0735-7036.117.2.176 12856788

[CR10] Blundell, C., Uria, B., Pritzel, A., Li, Y., Ruderman, A., Leibo, J. Z., Rae, J., Wierstra, D., & Hassabis, D. (2016). *Model-Free Episodic Control* (arXiv:1606.04460). arXiv. 10.48550/arXiv.1606.04460

[CR11] Boeckle, M., Schiestl, M., Frohnwieser, A., Gruber, R., Miller, R., Suddendorf, T., Gray, R. D., Taylor, A. H., & Clayton, N. S. (2020). New Caledonian crows plan for specific future tool use. *Proceedings of the Royal Society b: Biological Sciences,**287*(1938), 20201490. 10.1098/rspb.2020.1490 10.1098/rspb.2020.1490 PMC7735258 33143583

[CR12] Boyer, P. (2008). Evolutionary economics of mental time travel? *Trends in Cognitive Sciences,**12*(6), 219–224. 10.1016/j.tics.2008.03.003 18468941 10.1016/j.tics.2008.03.003

[CR13] Boyle, A. (2019). Learning From the Past: Epistemic Generativity and the Function of Episodic Memory. *Journal of Consciousness Studies,**26*(5–6), 242–251.

[CR14] Boyle, A. (2020). The impure phenomenology of episodic memory. *Mind & Language,**35*(5), 641–660. 10.1111/mila.12261

[CR15] Boyle, A. (2021). The mnemonic functions of episodic memory. *Philosophical Psychology*, *0*(0), 1–23. 10.1080/09515089.2021.1980520

[CR16] Boyle, A., & Blomkvist, A. (2024). Elements of episodic memory: Insights from artificial agents. *Philosophical Transactions of the Royal Society b: Biological Sciences.*10.1098/rstb.2023.0416 10.1098/rstb.2023.0416 PMC11449156 39278254

[CR17] Boyle, A., & Brown, S. A. B. (In Prep.). *Episodic Memory in Animals*. ttps://philsci-archive.pitt.edu/id/eprint/23782

[CR18] Bräuer, J., & Call, J. (2015). Apes produce tools for future use. *American Journal of Primatology,**77*(3), 254–263. 10.1002/ajp.22341 25236323 10.1002/ajp.22341

[CR19] Brea, J., Clayton, N. S., & Gerstner, W. (2023). Computational models of episodic-like memory in food-caching birds. *Nature Communications,**14*(1), 2979. 10.1038/s41467-023-38570-x 37221167 10.1038/s41467-023-38570-x PMC10205804

[CR20] Brown, S. A. B. (2023). Inter-temporal rationality without temporal representation. *Mind & Language,**38*(2), 495–514. 10.1111/mila.12405

[CR21] Brown, S. A. B. (2024). Episodic Memory and Unrestricted Learning. *Philosophy of Science,**91*(1), 90–110. 10.1017/psa.2023.16

[CR22] Cartwright, B. A., & Collett, T. S. (1987). Landmark maps for honeybees. *Biological Cybernetics,**57*(1), 85–93. 10.1007/BF00318718

[CR23] Cheng, S., & Werning, M. (2016). What is episodic memory if it is a natural kind? *Synthese,**193*(5), 1345–1385. 10.1007/s11229-014-0628-6

[CR24] Chettih, S. N., Mackevicius, E. L., Hale, S., & Aronov, D. (2024). Barcoding of episodic memories in the hippocampus of a food-caching bird. *Cell,**187*(8), 1922-1935.e20. 10.1016/j.cell.2024.02.032 38554707 10.1016/j.cell.2024.02.032 PMC11015962

[CR25] Clayton, N. S. (1999). What animals remember about past events: An ethological approach. In *Techniques in the Behavioral and Neural Sciences* (Vol. 13, pp. 614–626). Elsevier. https://www.sciencedirect.com/science/article/pii/S0921070999800480

[CR26] Clayton, N. S., & Dickinson, A. (1998). Episodic-like memory during cache recovery by scrub jays. *Nature*, *395*(6699), Article 6699. 10.1038/26216 10.1038/26216 9751053

[CR27] Clayton, N. S., Griffiths, D. P., Emery, N. J., & Dickinson, A. (2001). Elements of episodic-like memory in animals. *Philosophical Transactions of the Royal Society of London. Series B*, *356*(1413), 1483–1491. 10.1098/rstb.2001.0947 10.1098/rstb.2001.0947 PMC1088530 11571038

[CR28] Clayton, N. S., & Krebs, J. R. (1994). One-trial associative memory: Comparison of food-storing and nonstoring species of birds. *Animal Learning & Behavior,**22*(4), 366–372. 10.3758/BF03209155

[CR29] Clayton, N. S., Yu, K. S., & Dickinson, A. (2003). Interacting cache memories: Evidence for flexible memory use by Western scrub-jays (Aphelocoma californica). *Journal of Experimental Psychology: Animal Behavior Processes,**29*(1), 14–22. 10.1037/0097-7403.29.1.14 12561130

[CR30] Clewett, D., DuBrow, S., & Davachi, L. (2019). Transcending time in the brain: How event memories are constructed from experience. *Hippocampus,**29*(3), 162–183. 10.1002/hipo.23074 30734391 10.1002/hipo.23074 PMC6629464

[CR31] Correia, S. P. C., Dickinson, A., & Clayton, N. S. (2007). Western scrub-jays anticipate future needs independently of their current motivational state. *Current Biology: CB,**17*(10), 856–861. 10.1016/j.cub.2007.03.063 17462894 10.1016/j.cub.2007.03.063

[CR32] Cosmides, L., & Tooby, J. (2000). Consider the Source: The Evolution of Adaptations for Decoupling and Metarepresentation. In D. Sperber (Ed.), *Metarepresentations: A Multidisciplinary Perspective* (p. 0). Oxford University Press. 10.1093/oso/9780195141146.003.0004

[CR33] Craver, C. F. (2001). Role Functions, Mechanisms, and Hierarchy. *Philosophy of Science,**68*(1), 53–74.

[CR34] Crystal, J. D. (2021). Evaluating evidence from animal models of episodic memory. *Journal of Experimental Psychology: Animal Learning and Cognition,**47*(3), 337–356. 10.1037/xan0000294 34618532 10.1037/xan0000294

[CR35] Crystal, J. D., Alford, W. T., Zhou, W., & Hohmann, A. G. (2013). Source memory in the rat. *Current Biology,**23*(5), 387–391. 10.1016/j.cub.2013.01.023 23394830 10.1016/j.cub.2013.01.023 PMC3595394

[CR36] Cummins, R. E. (1975). Functional Analysis. *Journal of Philosophy,**72*(November), 741–764. 10.2307/2024640

[CR37] Currie, A. (2021). Comparative Thinking in Biology. *Cambridge University Press*. 10.1017/9781108616683

[CR38] Dally, J. M., Emery, N. J., & Clayton, N. S. (2009). Avian theory of mind and counter espionage by food-caching western scrub-jays (Aphelocoma californica). *European Journal of Developmental Psychology,**7*(1), 17–37. 10.1080/17405620802571711

[CR39] Davidson, P. S., Drouin, H., Kwan, D., Moscovitch, M., & Rosenbaum, R. S. (2012). Memory as Social Glue: Close Interpersonal Relationships in Amnesic Patients. *Frontiers in Psychology*, *3*. 10.3389/fpsyg.2012.00531 10.3389/fpsyg.2012.00531 PMC3541054 23316176

[CR40] Davies, J. R., & Clayton, N. (2024). Is episodic-like memory like episodic memory. *Philosophical Transactions of the Royal Society b: Biological Sciences.*10.1098/rstb.2023.0397 10.1098/rstb.2023.0397 PMC11449162 39278246

[CR41] Davies, J. R., Garcia-Pelegrin, E., Baciadonna, L., Pilenga, C., Favaro, L., & Clayton, N. S. (2022). Episodic-like memory in common bottlenose dolphins. *Current Biology*, S0960982222009915. 10.1016/j.cub.2022.06.032 10.1016/j.cub.2022.06.032 35882234

[CR42] Davies, J. R., Garcia-Pelegrin, E., & Clayton, N. S. (2024a). Eurasian jays (Garrulus glandarius) show episodic-like memory through the incidental encoding of information. *PLoS ONE,**19*(5), e0301298. 10.1371/journal.pone.0301298 38748646 10.1371/journal.pone.0301298 PMC11095760

[CR43] Davies, J. R., Keuneke, L. S., Clayton, N. S., & Davidson, G. L. (2024b). Episodic-like memory in wild free-living blue tits and great tits. *Current Biology,**34*(16), 3593-3602.e5. 10.1016/j.cub.2024.06.029 38964317 10.1016/j.cub.2024.06.029

[CR44] De Brigard, F. (2014). Is memory for remembering? Recollection as a form of episodic hypothetical thinking. *Synthese,**191*(2), 155–185. 10.1007/s11229-013-0247-7

[CR45] De Brigard, F., & Parikh, N. (2019). Episodic Counterfactual Thinking. *Current Directions in Psychological Science,**28*(1), 59–66. 10.1177/0963721418806512

[CR46] de Kort, S. R., Dickinson, A., & Clayton, N. S. (2005). Retrospective cognition by food-caching western scrub-jays. *Learning and Motivation,**36*(2), 159–176. 10.1016/j.lmot.2005.02.008

[CR47] Deese, J. (1959). On the prediction of occurrence of particular verbal intrusions in immediate recall. *Journal of Experimental Psychology,**58*(1), 17–22. 10.1037/h0046671 13664879 10.1037/h0046671

[CR48] Dehaene, S., Cohen, L., Morais, J., & Kolinsky, R. (2015). Illiterate to literate: Behavioural and cerebral changes induced by reading acquisition. *Nature Reviews Neuroscience,**16*(4), 234–244. 10.1038/nrn3924 25783611 10.1038/nrn3924

[CR49] Eichenbaum, H. (2017a). Prefrontal–hippocampal interactions in episodic memory. *Nature Reviews Neuroscience,**18*(9), 547–558. 10.1038/nrn.2017.74 28655882 10.1038/nrn.2017.74

[CR50] Eichenbaum, H. (2017b). The role of the hippocampus in navigation is memory. *Journal of Neurophysiology,**117*(4), 1785–1796. 10.1152/jn.00005.2017 28148640 10.1152/jn.00005.2017 PMC5384971

[CR51] Eichenbaum, H., Fortin, N. J., Ergorul, C., Wright, S. P., & Agster, K. L. (2005). Episodic recollection in animals: “If it walks like a duck and quacks like a duck….” *Learning and Motivation,**36*(2), 190–207. 10.1016/j.lmot.2005.02.006

[CR52] Feeney, M. C., Roberts, W. A., & Sherry, D. F. (2009). Memory for what, where, and when in the black-capped chickadee (Poecile atricapillus). *Animal Cognition,**12*(6), 767–777. 10.1007/s10071-009-0236-x 19466468 10.1007/s10071-009-0236-x

[CR53] Fugazza, C., Pogány, Á., & Miklósi, Á. (2016). Recall of Others’ Actions after Incidental Encoding Reveals Episodic-like Memory in Dogs. *Current Biology,**26*(23), 3209–3213. 10.1016/j.cub.2016.09.057 27889264 10.1016/j.cub.2016.09.057

[CR54] Garson, J. (2012). Function, selection, and construction in the brain. *Synthese,**189*(3), 451–481. 10.1007/s11229-012-0122-y

[CR55] Gershman, S. J., & Daw, N. D. (2017). Reinforcement learning and episodic memory in humans and animals: An integrative framework. *Annual Review of Psychology,**68*, 101–128. 10.1146/annurev-psych-122414-033625 27618944 10.1146/annurev-psych-122414-033625 PMC5953519

[CR56] Godfrey-Smith, P. (1994). A Modern History Theory of Functions. *Noûs,**28*(3), 344–362. 10.2307/2216063

[CR57] Gould, J. L. (1986). The locale map of honey bees: Do insects have cognitive maps? *Science (New York, N.Y.)*, *232*(4752). 10.1126/science.232.4752.861 10.1126/science.232.4752.861 17755968

[CR58] Gould, S. J., & Lewontin, R. C. (1979). The spandrels of San Marco and the Panglossian paradigm: A critique of the adaptationist programme. *Proceedings of the Royal Society of London. Series B. Biological Sciences*, *205*, 581–598. 10.1098/rspb.1979.0086 42062

[CR59] Halina, M. (2023). Methods in Comparative Cognition. In E. N. Zalta & U. Nodelman (Eds.), *The Stanford Encyclopedia of Philosophy* (Fall 2023). Metaphysics Research Lab, Stanford University. https://plato.stanford.edu/archives/fall2023/entries/comparative-cognition/

[CR60] Hamilton, T. J., Myggland, A., Duperreault, E., May, Z., Gallup, J., Powell, R. A., Schalomon, M., & Digweed, S. M. (2016). Episodic-like memory in zebrafish. *Animal Cognition,**19*(6), 1071–1079. 10.1007/s10071-016-1014-1 27421709 10.1007/s10071-016-1014-1

[CR61] Harten, L., Chen, X., de Marcas, L., Rachum, A., Handel, M., Goldshtein, A., Levi, M. F., Rosencwaig, S., & Yovel, Y. (2024). Time-mapping and future-oriented behavior in free-ranging wild fruit bats. *Current Biology: CB,**34*(13), 3005-3010.e4. 10.1016/j.cub.2024.05.046 38906144 10.1016/j.cub.2024.05.046

[CR62] Hassabis, D., Kumaran, D., Vann, S. D., & Maguire, E. A. (2007). Patients with hippocampal amnesia cannot imagine new experiences. *Proceedings of the National Academy of Sciences of the United States of America,**104*(5), 1726–1731. 10.1073/pnas.0610561104 17229836 10.1073/pnas.0610561104 PMC1773058

[CR63] Healy, S. D., & Hurly, T. A. (2013). What hummingbirds can tell us about cognition in the wild. *Comparative Cognition & Behavior Reviews*, *8*. https://comparative-cognition-and-behavior-reviews.org/vol8_healy_hurly/

[CR64] Henke, K. (2010). A model for memory systems based on processing modes rather than consciousness. *Nature Reviews. Neuroscience,**11*(7), 523–532. 10.1038/nrn2850 20531422 10.1038/nrn2850

[CR65] Heyes, C. M. (2018). Précis of Cognitive Gadgets: The Cultural Evolution of Thinking. *Behavioral and Brain Sciences,**42*(e169), 1–58. 10.1017/S0140525X18002145

[CR66] Hoerl, C., & McCormack, T. (2016). Making Decisions About the Future: Regret and the Cognitive Function of Episodic Memory. In K. Michaelian, S. Klein, & K. Szpunar (Eds.), *Seeing the future: Theoretical perspectives on future-oriented mental time travel* (pp. 241–266). Oxford University Press.

[CR67] Hoerl, C., & McCormack, T. (2017). Animal Minds in Time: The Question of Episodic Memory. In K. Andrews & J. Beck (Eds.), *The Routledge Handbook of Philosophy of Animal Minds* (pp. 56–64). Routledge.

[CR68] Hoerl, C., & McCormack, T. (2019). Thinking in and about time: A dual systems perspective on temporal cognition. *Behavioral and Brain Sciences,**42*, e244. 10.1017/S0140525X18002157 10.1017/S0140525X18002157 30251619

[CR69] Huber, D. E. (2024). The Primary Function of MTL is Memory, not Navigation: Grid Cells are Non-spatial (what) and Place Cells are Memories (what and where) that Cause Grid Fields through Retrieval. *eLife*, *13*. 10.7554/eLife.95733.1

[CR70] Hume, D. (1748). *An Enquiry concerning Human Understanding* (P. Millican, Ed.). Oxford University Press.

[CR71] Janmaat, K. R. L., Byrne, R. W., & Zuberbühler, K. (2006a). Evidence for a spatial memory of fruiting states of rainforest trees in wild mangabeys. *Animal Behaviour,**72*(4), 797–807. 10.1016/j.anbehav.2005.12.009

[CR72] Janmaat, K. R. L., Byrne, R. W., & Zuberbühler, K. (2006b). Primates take weather into account when searching for fruits. *Current Biology: CB,**16*(12), 1232–1237. 10.1016/j.cub.2006.04.031 16782015 10.1016/j.cub.2006.04.031

[CR73] Jozet-Alves, C., Bertin, M., & Clayton, N. S. (2013). Evidence of episodic-like memory in cuttlefish. *Current Biology,**23*(23), R1033–R1035. 10.1016/j.cub.2013.10.021 24309275 10.1016/j.cub.2013.10.021

[CR74] Kaminski, J., Fischer, J., & Call, J. (2008). Prospective object search in dogs: Mixed evidence for knowledge of What and Where. *Animal Cognition,**11*(2), 367–371. 10.1007/s10071-007-0124-1 18060437 10.1007/s10071-007-0124-1 PMC2757617

[CR75] Keven, N. (2024). Can episodic memory deter cheating and promote altruism? *Synthese,**203*(5), 132. 10.1007/s11229-024-04560-9

[CR76] Kosaki, Y., Lin, T.-C.E., Horne, M. R., Pearce, J. M., & Gilroy, K. E. (2014). The role of the hippocampus in passive and active spatial learning. *Hippocampus,**24*(12), 1633–1652. 10.1002/hipo.22343 25131441 10.1002/hipo.22343 PMC4258078

[CR77] Kouwenberg, A.-L., Walsh, C. J., Morgan, B. E., & Martin, G. M. (2009). Episodic-like memory in crossbred Yucatan minipigs (Sus scrofa). *Applied Animal Behaviour Science,**117*(3), 165–172. 10.1016/j.applanim.2009.01.005

[CR78] Krebs, J. R., Sherry, D. F., Healy, S. D., Perry, V. H., & Vaccarino, A. L. (1989). Hippocampal specialization of food-storing birds. *Proceedings of the National Academy of Sciences of the United States of America,**86*(4), 1388–1392. 10.1073/pnas.86.4.1388 2919184 10.1073/pnas.86.4.1388 PMC286696

[CR79] Kurby, C. A., & Zacks, J. M. (2008). Segmentation in the perception and memory of events. *Trends in Cognitive Sciences,**12*(2), 72–79. 10.1016/j.tics.2007.11.004 18178125 10.1016/j.tics.2007.11.004 PMC2263140

[CR80] Kwan, D., Craver, C. F., Green, L., Myerson, J., & Rosenbaum, R. S. (2013). Dissociations in future thinking following hippocampal damage: Evidence from discounting and time perspective in episodic amnesia. *Journal of Experimental Psychology: General,**142*(4), 1355–1369. 10.1037/a0034001 23978187 10.1037/a0034001

[CR81] Lengyel, M., & Dayan, P. (2007). Hippocampal Contributions to Control: The Third Way. In J. Platt, D. Koller, Y. Singer, & S. Roweis (Eds.), *Advances in Neural Information Processing Systems* (Vol. 20). Curran Associates, Inc. https://proceedings.neurips.cc/paper/2007/file/1f4477bad7af3616c1f933a02bfabe4e-Paper.pdf. Accessed August 2024

[CR82] Liu, A. A., Henin, S., Abbaspoor, S., Bragin, A., Buffalo, E. A., Farrell, J. S., Foster, D. J., Frank, L. M., Gedankien, T., Gotman, J., Guidera, J. A., Hoffman, K. L., Jacobs, J., Kahana, M. J., Li, L., Liao, Z., Lin, J. J., Losonczy, A., Malach, R., … Buzsáki, G. (2022). A consensus statement on detection of hippocampal sharp wave ripples and differentiation from other fast oscillations. *Nature Communications*, *13*(1), 6000. 10.1038/s41467-022-33536-x 10.1038/s41467-022-33536-x PMC9556539 36224194

[CR83] Maguire, E. A., & Mullally, S. L. (2013). The Hippocampus: A Manifesto for Change. *Journal of Experimental Psychology. General,**142*(4), 1180–1189. 10.1037/a0033650 23855494 10.1037/a0033650 PMC3906798

[CR84] Mahr, J. B. (2022). Episodic memory: And what is it for? In *Current controversies in philosophy of memory* (pp. 149–165). Routledge. https://api.taylorfrancis.com/content/chapters/edit/download?identifierName=doi&identifierValue=10.4324/9781003002277-12&type=chapterpdf

[CR85] Mahr, J. B., & Csibra, G. (2018). Why do we remember? The communicative function of episodic memory. *Behavioral and Brain Sciences*, *41*. 10.1017/S0140525X17000012 10.1017/S0140525X17000012 PMC5404722 28100294

[CR86] Mahr, J. B., van Bergen, P., Sutton, J., Schacter, D. L., & Heyes, C. (2023). Mnemicity: A Cognitive Gadget? *Perspectives on Psychological Science,**18*(5), 1160–1177. 10.1177/17456916221141352 36649218 10.1177/17456916221141352 PMC11759954

[CR87] Martin, R. J., Martin, G. K., Roberts, W. A., & Sherry, D. F. (2021). No evidence for future planning in Canada jays (Perisoreus canadensis). *Biology Letters,**17*(12), 20210504. 10.1098/rsbl.2021.0504 34875182 10.1098/rsbl.2021.0504 PMC8651407

[CR88] Martin-Ordas, G., Schumacher, L., & Call, J. (2012). Sequential tool use in great apes. *PLoS ONE,**7*(12), e52074. 10.1371/journal.pone.0052074 23300592 10.1371/journal.pone.0052074 PMC3530559

[CR89] McClelland, J. L., McNaughton, B. L., & O’Reilly, R. C. (1995). Why there are complementary learning systems in the hippocampus and neocortex: Insights from the successes and failures of connectionist models of learning and memory. *Psychological Review,**102*(3), 419–457. 10.1037/0033-295X.102.3.419 7624455 10.1037/0033-295X.102.3.419

[CR90] Michaelian, K. (2016). *Mental Time Travel: Episodic Memory and Our Knowledge of the Personal Past*. MIT Press.

[CR91] Mnih, V., Kavukcuoglu, K., Silver, D., Rusu, A. A., Veness, J., Bellemare, M. G., Graves, A., Riedmiller, M., Fidjeland, A. K., Ostrovski, G., Petersen, S., Beattie, C., Sadik, A., Antonoglou, I., King, H., Kumaran, D., Wierstra, D., Legg, S., & Hassabis, D. (2015). Human-level control through deep reinforcement learning. *Nature,**518*(7540), 529–533. 10.1038/nature14236 25719670 10.1038/nature14236

[CR92] Moser, E. I., Kropff, E., & Moser, M.-B. (2008). Place cells, grid cells, and the brain’s spatial representation system. *Annual Review of Neuroscience,**31*, 69–89. 10.1146/annurev.neuro.31.061307.090723 18284371 10.1146/annurev.neuro.31.061307.090723

[CR93] Murray, E. A., Wise, S. P., & Graham, K. S. (2017). *The evolution of memory systems: Ancestors, anatomy, and adaptations*. Oxford University Press.

[CR94] Musgrave, S., Koni, D., Morgan, D., & Sanz, C. (2023). Planning abilities of wild chimpanzees (Pan troglodytes troglodytes) in tool-using contexts. *Primates*. 10.1007/s10329-023-01106-4 38103142 10.1007/s10329-023-01106-4 PMC11561005

[CR95] Nadel, L., & Moscovitch, M. (1998). Hippocampal contributions to cortical plasticity. *Neuropharmacology,**37*(4), 431–439. 10.1016/S0028-3908(98)00057-4 9704984 10.1016/s0028-3908(98)00057-4

[CR96] Neander, K. (1991). Functions as Selected Effects: The Conceptual Analyst’s Defense. *Philosophy of Science,**58*(2), 168–184.

[CR97] Neander, K. (2016). Biological function. In *Routledge Encyclopedia of Philosophy* (1st ed.). Routledge. 10.4324/9780415249126-Q140-1

[CR98] O’Kane, G., Kensinger, E. A., & Corkin, S. (2004). Evidence for semantic learning in profound amnesia: An investigation with patient H.M. *Hippocampus*, *14*(4), 417–425. 10.1002/hipo.20005 10.1002/hipo.20005 15224979

[CR99] O’Keefe, J. M., Nadel, L., & O’Keefe, J. (1978). *The hippocampus as a cognitive map*. Clarendon Press.

[CR100] Ólafsdóttir, H. F., Bush, D., & Barry, C. (2018). The Role of Hippocampal Replay in Memory and Planning. *Current Biology: CB,**28*(1), R37–R50. 10.1016/j.cub.2017.10.073 29316421 10.1016/j.cub.2017.10.073 PMC5847173

[CR101] Osvath, M. (2009). Spontaneous planning for future stone throwing by a male chimpanzee. *Current Biology: CB,**19*(5), R190-191. 10.1016/j.cub.2009.01.010 19278627 10.1016/j.cub.2009.01.010

[CR102] Osvath, M., & Karvonen, E. (2012). Spontaneous innovation for future deception in a male chimpanzee. *PLoS ONE,**7*(5), e36782. 10.1371/journal.pone.0036782 22590606 10.1371/journal.pone.0036782 PMC3348900

[CR103] Osvath, M., & Osvath, H. (2008). Chimpanzee (Pan troglodytes) and orangutan (Pongo abelii) forethought: Self-control and pre-experience in the face of future tool use. *Animal Cognition,**11*(4), 661–674. 10.1007/s10071-008-0157-0 18553113 10.1007/s10071-008-0157-0

[CR104] Panoz-Brown, D., Iyer, V., Carey, L. M., Sluka, C. M., Rajic, G., Kestenman, J., Gentry, M., Brotheridge, S., Somekh, I., Corbin, H. E., Tucker, K. G., Almeida, B., Hex, S. B., Garcia, K. D., Hohmann, A. G., & Crystal, J. D. (2018). Replay of Episodic Memories in the Rat. *Current Biology: CB,**28*(10), 1628-1634.e7. 10.1016/j.cub.2018.04.006 29754898 10.1016/j.cub.2018.04.006 PMC5964044

[CR105] Puddifoot, K., & Bortolotti, L. (2019). Epistemic innocence and the production of false memory beliefs. *Philosophical Studies: An International Journal for Philosophy in the Analytic Tradition,**176*(3), 755–780. 10.1007/s11098-018-1038-2 PMC7618592 41503522

[CR106] Raby, C. R., Alexis, D. M., Dickinson, A., & Clayton, N. S. (2007). Planning for the future by western scrub-jays. *Nature*, *445*(7130), Article 7130. 10.1038/nature05575 10.1038/nature05575 17314979

[CR107] Raby, C. R., & Clayton, N. S. (2009). Prospective cognition in animals. *Behavioural Processes,**80*(3), 314–324. 10.1016/j.beproc.2008.12.005 20522320 10.1016/j.beproc.2008.12.005

[CR108] Rattenborg, N. C., & Martinez-Gonzalez, D. (2011). A bird-brain view of episodic memory. *Behavioural Brain Research,**222*(1), 236–245. 10.1016/j.bbr.2011.03.030 21420438 10.1016/j.bbr.2011.03.030

[CR109] Redshaw, J., & Bulley, A. (2018). Future‑thinking in animals: Capacities and limits. In *The psychology of thinking about the future* (pp. 31–51). The Guilford Press.

[CR110] Robins, S. K. (2022). Episodic memory is not for the future. In *Current Controversies in Philosophy of Memory*. Routledge.

[CR111] Roediger, H. L., & McDermott, K. B. (1995). Creating false memories: Remembering words not presented in lists. *Journal of Experimental Psychology: Learning, Memory, and Cognition,**21*(4), 803–814. 10.1037/0278-7393.21.4.803

[CR112] Roediger, H. L., III. (1996). Memory Illusions. *Journal of Memory and Language,**35*(2), 76–100. 10.1006/jmla.1996.0005

[CR113] Rosenbaum, R. S., Köhler, S., Schacter, D. L., Moscovitch, M., Westmacott, R., Black, S. E., Gao, F., & Tulving, E. (2005). The case of K.C.: Contributions of a memory-impaired person to memory theory. *Neuropsychologia*, *43*(7), 989–1021. 10.1016/j.neuropsychologia.2004.10.007 10.1016/j.neuropsychologia.2004.10.007 15769487

[CR114] Sammons, R. P., Vezir, M., Moreno-Velasquez, L., Cano, G., Orlando, M., Sievers, M., Grasso, E., Metodieva, V. D., Kempter, R., Schmidt, H., & Schmitz, D. (2024). Structure and function of the hippocampal CA3 module. *Proceedings of the National Academy of Sciences,**121*(6), e2312281120. 10.1073/pnas.2312281120 10.1073/pnas.2312281120 PMC10861929 38289953

[CR115] Sato, N. (2021). Episodic-like memory of rats as retrospective retrieval of incidentally encoded locations and involvement of the retrosplenial cortex. *Scientific Reports,**11*(1), 2217. 10.1038/s41598-021-81943-9 33500512 10.1038/s41598-021-81943-9 PMC7838390

[CR116] Schacter, D. L. (1999). The seven sins of memory: Insights from psychology and cognitive neuroscience. *American Psychologist,**54*(3), 182–203. 10.1037/0003-066X.54.3.182 10199218 10.1037//0003-066x.54.3.182

[CR117] Schacter, D. L., & Addis, D. R. (2007). The cognitive neuroscience of constructive memory: Remembering the past and imagining the future. *Philosophical Transactions of the Royal Society b: Biological Sciences,**362*, 773–786. 10.1098/rstb.2007.2087 10.1098/rstb.2007.2087 PMC2429996 17395575

[CR118] Schacter, D. L., Addis, D. R., & Buckner, R. L. (2007). Remembering the past to imagine the future: The prospective brain. *Nature Reviews Neuroscience*, *8*(9), Article 9. 10.1038/nrn2213 10.1038/nrn2213 17700624

[CR119] Schacter, D. L., Benoit, R. G., De Brigard, F., & Szpunar, K. K. (2015). Episodic future thinking and episodic counterfactual thinking: Intersections between memory and decisions. *Neurobiology of Learning and Memory,**117*, 14–21. 24373942 10.1016/j.nlm.2013.12.008 PMC4071128

[CR120] Schacter, D. L., Carpenter, A. C., Devitt, A., Roberts, R. P., & Addis, D. R. (2018). Constructive episodic simulation, flexible recombination, and memory errors. *Behavioral and Brain Sciences*, *41*. 10.1017/S0140525X17001510 10.1017/S0140525X17001510 29353590

[CR121] Schacter, D. L., Guerin, S. A., & Jacques, P. L. S. (2011). Memory distortion: An adaptive perspective. *Trends in Cognitive Sciences,**15*(10), 467–474. 10.1016/j.tics.2011.08.004 21908231 10.1016/j.tics.2011.08.004 PMC3183109

[CR122] Schnell, A. K., Amodio, P., Boeckle, M., & Clayton, N. S. (2021a). How intelligent is a cephalopod? Lessons from comparative cognition. *Biological reviews of the Cambridge Philosophical Society,**96*(1), 162–178. 10.1111/brv.12651 32893443 10.1111/brv.12651

[CR123] Schnell, A. K., Clayton, N. S., Hanlon, R. T., & Jozet-Alves, C. (2021b). Episodic-like memory is preserved with age in cuttlefish. *Proceedings. Biological Sciences,**288*(1957), 20211052. 10.1098/rspb.2021.1052 34403629 10.1098/rspb.2021.1052 PMC8370807

[CR124] Schulz, A. W., & Robins, S. (2022). Episodic Memory, Simulated Future Planning, and Their Evolution. *Review of Philosophy and Psychology*, 1–22. 10.1007/s13164-021-00601-1

[CR125] Schwartz, A. (2020). Simulationism and the Function(s) of Episodic Memory. *Review of Philosophy and Psychology,**11*(2), 487–505. 10.1007/s13164-020-00461-1

[CR126] Shea, N. (2018). *Representation in Cognitive Science*. Oxford University Press.

[CR127] Sherry, D. F. (1984). Food storage by black-capped chickadees: Memory for the location and contents of caches. *Animal Behaviour,**32*(2), 451–464. 10.1016/S0003-3472(84)80281-X

[CR128] Sherry, D. F. (2011). The hippocampus of food-storing birds. *Brain, Behavior and Evolution,**78*(2), 133–135. 10.1159/000330314 21876337 10.1159/000330314

[CR129] Sherry, D. F., & Vaccarino, A. L. (1989). Hippocampus and memory for food caches in black-capped chickadees. *Behavioral Neuroscience,**103*(2), 308–318. 10.1037/0735-7044.103.2.308

[CR130] Sherry, D. F., Vaccarino, A. L., Buckenham, K., & Herz, R. S. (1989). The hippocampal complex of food-storing birds. *Brain, Behavior and Evolution,**34*(5), 308–317. 10.1159/000116516 2611638 10.1159/000116516

[CR131] Sober, E. (2005). Comparative psychology meets evolutionary biology: Morgan’s canon and cladistic parsimony. *Thinking with Animals: New Perspectives on Anthropomorphism*, 85–99.

[CR132] Sober, E. (2009). Parsimony and Models of Animal Minds. In R. W. Lurz (Ed.), *The Philosophy of Animal Minds* (p. 237). Cambridge University Press.

[CR133] Sober, E. (2015). *Ockham’s Razors: A User’s Manual*. Cambridge University Press.

[CR134] Spanò, G., Pizzamiglio, G., McCormick, C., Clark, I. A., De Felice, S., Miller, T. D., Edgin, J. O., Rosenthal, C. R., & Maguire, E. A. (2020). *Dreaming with Hippocampal Damage. Elife,**9*, e56211. 10.7554/eLife.56211 32508305 10.7554/eLife.56211 PMC7279885

[CR135] Stotz, K. (2014). Extended evolutionary psychology: The importance of transgenerational developmental plasticity. *Frontiers in Psychology*, *5*. 10.3389/fpsyg.2014.00908 10.3389/fpsyg.2014.00908 PMC4138557 25191292

[CR136] Suddendorf, T., & Corballis, M. C. (1997). Mental time travel and the evolution of the human mind. *Genetic Social and General Psychology Monographs,**123*, 133–167. 9204544

[CR137] Suddendorf, T., & Corballis, M. C. (2007). The evolution of foresight: What is mental time travel, and is it unique to humans? *Behavioral and Brain Sciences,**30*(3), 299–313. 10.1017/S0140525X07001975 17963565 10.1017/S0140525X07001975

[CR138] Templer, V. L., & Hampton, R. R. (2013). Episodic memory in nonhuman animals. *Current Biology : CB,**23*(17), R801–R806. 10.1016/j.cub.2013.07.016 24028963 10.1016/j.cub.2013.07.016 PMC3799964

[CR139] Tulving, E. (2005). Episodic Memory and Autonoesis: Uniquely Human? In *The Missing Link in Cognition*. Oxford University Press. 10.1093/acprof:oso/9780195161564.003.0001

[CR140] Van Horik, J. O., Clayton, N. S., & Emery, N. J. (2012). Convergent Evolution of Cognition in Corvids, Apes and Other Animals. In T. K. Shackelford & J. Vonk (Eds.), *The Oxford Handbook of Comparative Evolutionary Psychology* (p. 0). Oxford University Press. 10.1093/oxfordhb/9780199738182.013.0005

[CR141] Van Leeuwen, N. (2013). The Meanings of “Imagine” Part I: Constructive Imagination. *Philosophy Compass,**8*(3), 220–230. 10.1111/j.1747-9991.2012.00508.x

[CR142] Wiltgen, B. J., Sanders, M. J., Anagnostaras, S. G., Sage, J. R., & Fanselow, M. S. (2006). Context Fear Learning in the Absence of the Hippocampus. *Journal of Neuroscience,**26*(20), 5484–5491. 10.1523/JNEUROSCI.2685-05.2006 16707800 10.1523/JNEUROSCI.2685-05.2006 PMC6675287

[CR143] Wimpenny, J. H., Weir, A. A. S., Clayton, L., Rutz, C., & Kacelnik, A. (2009). Cognitive Processes Associated with Sequential Tool Use in New Caledonian Crows. *PLoS ONE,**4*(8), e6471. 10.1371/journal.pone.0006471 19654861 10.1371/journal.pone.0006471 PMC2714693

[CR144] Ye, J., Ding, Q., Cui, J., Liu, Z., Jia, L., Qin, X., Xu, H., & Wang, Y. (2022). A meta-analysis of the effects of episodic future thinking on delay discounting. *Quarterly Journal of Experimental Psychology,**75*(10), 1876–1891. 10.1177/17470218211066282 10.1177/17470218211066282 34841982

[CR145] Zacks, O., Ginsburg, S., & Jablonka, E. (2022). The Futures of the Past The Evolution of Imaginative Animals. *Journal of Consciousness Studies*, *29*(3–4), 29–61. 10.53765/20512201.29.3.029

[CR146] Zacks, O., & Jablonka, E. (2023). The evolutionary origins of the Global Neuronal Workspace in vertebrates. *Neuroscience of Consciousness*, *2023*(1), niad020. 10.1093/nc/niad020 10.1093/nc/niad020 PMC10499063 37711313

[CR147] Zeng, X., Diekmann, N., Wiskott, L., & Cheng, S. (2023). Modeling the function of episodic memory in spatial learning. *Frontiers in Psychology*, *14*. https://www.frontiersin.org/articles/10.3389/fpsyg.2023.1160648 10.3389/fpsyg.2023.1160648 PMC10149844 37138984

[CR148] Zentall, T. R., Clement, T. S., Bhatt, R. S., & Allen, J. (2001). Episodic-like memory in pigeons. *Psychonomic Bulletin & Review,**8*(4), 685–690. 10.3758/BF03196204 11848586 10.3758/bf03196204

[CR149] Zentall, T. R., Singer, R. A., & Stagner, J. P. (2008). Episodic-like memory: Pigeons can report location pecked when unexpectedly asked. *Behavioural Processes,**79*(2), 93–98. 10.1016/j.beproc.2008.05.003 18602224 10.1016/j.beproc.2008.05.003

[CR150] Zhou, W., & Crystal, J. D. (2009). Evidence for remembering when events occurred in a rodent model of episodic memory. *Proceedings of the National Academy of Sciences,**106*(23), 9525–9529. 10.1073/pnas.0904360106 10.1073/pnas.0904360106 PMC2695044 19458264

[CR151] Zhou, W., Hohmann, A. G., & Crystal, J. D. (2012). Rats answer an unexpected question after incidental encoding. *Current Biology: CB,**22*(12), 1149–1153. 10.1016/j.cub.2012.04.040 22633809 10.1016/j.cub.2012.04.040 PMC3376895

[CR152] Zinkivskay, A., Nazir, F., & Smulders, T. V. (2009). What-where-when memory in magpies (Pica pica). *Animal Cognition,**12*(1), 119–125. 10.1007/s10071-008-0176-x 18670793 10.1007/s10071-008-0176-x

